# IDconverter and IDClight: Conversion and annotation of gene and protein IDs

**DOI:** 10.1186/1471-2105-8-9

**Published:** 2007-01-10

**Authors:** Andreu Alibés, Patricio Yankilevich, Andrés Cañada, Ramón Díaz-Uriarte

**Affiliations:** 1 Structural Biology and Biocomputing Programme, Centro Nacional de Investigaciones Oncológicas (CNIO), Melchor Fernández Almagro 3, 28029, Madrid, Spain

## Abstract

**Background:**

Researchers involved in the annotation of large numbers of gene, clone or protein identifiers are usually required to perform a one-by-one conversion for each identifier. When the field of research is one such as microarray experiments, this number may be around 30,000.

**Results:**

To help researchers map accession numbers and identifiers among clones, genes, proteins and chromosomal positions, we have designed and developed IDconverter and IDClight. They are two user-friendly, freely available web server applications that also provide additional functional information by mapping the identifiers on to pathways, Gene Ontology terms, and literature references. Both tools are high-throughput oriented and include identifiers for the most common genomic databases. These tools have been compared to other similar tools, showing that they are among the fastest and the most up-to-date.

**Conclusion:**

These tools provide a fast and intuitive way of enriching the information coming out of high-throughput experiments like microarrays. They can be valuable both to wet-lab researchers and to bioinformaticians.

## Background

The databases that can help annotate our data are fast changing and rapidly increasing in number. This makes it more difficult to easily integrate this useful information to the output from the different types of high-throughput experiments and analyses of those experiments. But integrating the available data is crucial to gain further biological understanding of our results [1].

Output from microarray and other high-throughput experiments involves several thousands of gene, clone or protein identifiers. Comparing results from different experiments with different identifiers may require a non trivial and tedious conversion of IDs one by one. Also, collecting functional information for several thousand identifiers from different data sources may end up being too time consuming a task. Motivated by the necessity of researchers at our Institute to obtain this information in a reliable, up-to-date and easy manner, we developed IDconverter and IDClight, two complementary applications that retrieve data from the same source, but present it in different ways to cover different users' needs.

Conversions between gene and protein identifiers may be one-to-one or one-to-many. For instance, a user working with a given gene (e.g., *p53*), and starting from its HUGO name (TP53), may be interested in knowing the corresponding Ensembl gene ID (ENSG00000141510), its UniGene cluster ID (Hs.408312), but also the cDNA clones from GenBank that map to it ([GenBank:AF052180], [GenBank:NM_000546], [GenBank:AY429684], etc.), the KEGG pathways where it is involved (hsa04010, hsa04110, hsa04210, etc.) or whether any protein produced by this gene has been solved and its 3D structure is in the Protein Data Bank ([PDB:1A1U], [PDB:1AIE], [PDB:1C26], etc.). With the tools here presented, all this information and more is readily available with a simple query through an intuitive user interface.

There are some tools available with similar objectives (Resourcerer [2], Onto-Translate [3], SOURCE [4], MatchMiner [5], GeneMerge [6], and AnnBuilder [7]) which provide mappings between some identifiers, but in general they lack a link between two of the most used databases: those at NCBI [8] and Ensembl [9]. In the Results section a comparison between some of these tools and the ones presented in this article is shown and discussed.

IDconverter and IDClight are integrated within Asterias [10], a new suite of tools for the analysis of genomic data. Output from all the applications on this suite can be enriched by adding a link to IDClight, provided that the user specifies the organism and type of identifier used. IDClight can also be linked from other external applications.

## Implementation

### Data

Both IDconverter and IDClight use data from a set of tables where, given our selection of databases, all possible conversions have already been pregenerated to ensure a quick answer to any query. This factor is specially important if we consider that some users need to convert the tens of thousands of gene IDs from a microarray experiment. Currently, the data in these pregenerated tables come from six different and publicly available databases: Ensembl [9], NCBI (UniGene and PubMed) [8], human chromosomal location from UCSC Genome Browser [11], KEGG pathway [12], and Reactome [13]. The UniGene database is only available in a plain text format, requiring its transformation into a MySQL database to pregenerate the tables.

**Figure 1:**
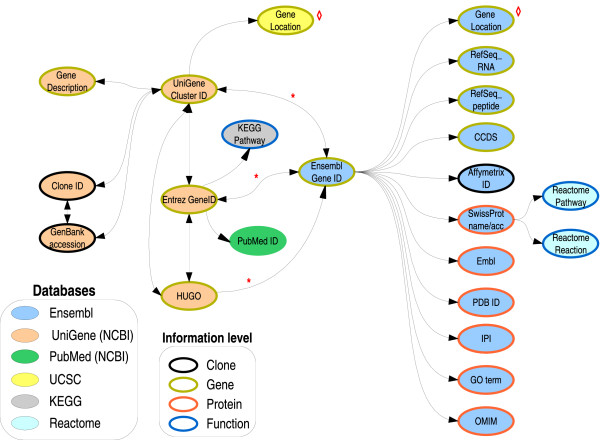
**Relationships between the different identifiers**. All relationships between identifiers that we have taken into account are displayed. The different identifiers are color coded according to which database they are taken from. The path taken from an identifier to another is always the shortest one. Red asterisk: To ensure that pregenerated information is as complete as possible, there are several paths to go from identifiers in the UniGene database to Ensembl Gene ID. The script first tries to map the Entrez Gene ID with an Ensembl Gene ID. If this fails, it tries with a UniGene Cluster ID, and finally with the HUGO name. Red diamond: Gene location is taken from either Ensembl or UCSC, or both, at user's wish.

In Figure 1 the different paths from and to all the different identifiers are shown. Except in those cases shown in the Figure, the shortest path between identifiers is always used, because it is the one that is likely to recover more information as the possibility of a missing identifier in the path is lower. The data is available for Human, Mouse and Rat, but more organisms could be easily added if they were interesting to the scientific community.

### Code

IDconverter front end is coded in PHP and IDClight is written in Python and uses the Apache module mod_python. Both applications run on an Apache web server and the databases are stored in a MySQL database server. To speed up the net waiting time from both tools, tables that store all possible conversions for each type of input identifier are periodically pregenerated (See additional file 1: Database schema for a brief description of the schema of the database where the pregenerated data is stored). IDconverter and IDClight run on two load-balanced servers with 2 dual core AMD processors each.

## Results

### IDconverter

IDconverter is the application that allows the user to map in batch mode multiple identifiers and select which output (types of identifiers and format) she prefers. The input identifiers can be gene names (HUGO), GenBank accessions, UniGene cluster IDs, Ensembl gene IDs, Clone IDs, Affymetrix IDs, RefSeq RNAs, RefSeq peptides, Entrez genes IDs, and SwissProt names.

Special attention was paid when designing a user friendly Graphical User Interface, suitable for biologists. This interface design makes the tool easy to understand and use, which results in greater user acceptance and facilitates the usage and incorporation in the workflow of a microarray laboratory.

**Figure 2:**
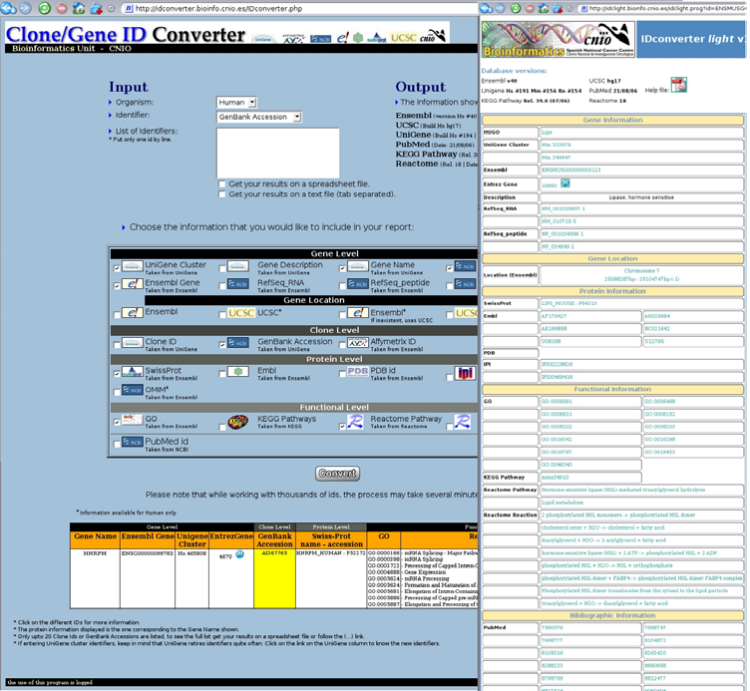
**Snapshot of IDconverter and IDClight**. On the left, IDconverter HTML output for a single human GenBank Accession. On the right, IDClight output for a mouse Ensembl gene.

There are 25 possible types of output identifiers and functional information that can be selected. The user can also select the format of the output information: an HTML table, a tab-delimited text file and an Excel spreadsheet file. If the HTML output is preferred, hyper links from each identifier to the original database are provided, as well as a link to iHOP [14], a gene network for navigating the literature. It must be noted that the chromosomal location of human genes is taken from two different sources: Ensembl and UCSC Genome Browser. The application allows the user to select the source of location information and can also complement one of the sources with the other one if information is missing. An example of the output is shown in Figure 2. IDconverter has been available for more than two years and it is currently receiving more than 100 requests per day.

### IDClight

IDClight is a tool created as a light and fast web service to be used to enrich the output of other data analysis tools. It is designed to be easily linkable from any application as all the input information (ID, ID type and organism) is sent to the application as parameters in the URL. The same 10 identifiers that can be used with IDconverter are allowed as input in this application. All possible output identifiers and functional and bibliographic information available are then displayed, with the appropriate hyper links from each identifier.

We would like to stress that IDClight can be trivially linked from external applications, as it is being done by our tools SignS, Tnasas, GeneSrF, Pomelo II and ADaCGH, available from the Asterias suite [10]. As an example, the URL to IDClight for the snapshot in Figure 2, looking for information on the mouse Ensembl gene ENSMUSG00000003123, is [15].

IDClight was launched on January 2006 and has been performing an average of 800 conversions per day.

### Comparison with other similar tools

Several of the tools with similar objectives that have been presented in the Background section have been compared to IDconverter. Those selected were MatchMiner [5], SOURCE [4], and Onto-Translate [3], because they present features most similar to the IDconverter ones. Other tools have not been so throughly reviewed in detail for different reasons: Resourcerer [2] is a useful tool provided that the user wants to use as input one of the commonly used microarrays, but it is not possible to use with a list of identifiers chosen by the user; GeneMerge [6] has not been considered in this comparison because its choice of input and output identifiers is very limited; finally, AnnBuilder [7] is a very interesting R package, but it is not an application in itself and requires installing R and Bioconductor.

**Figure 3:**
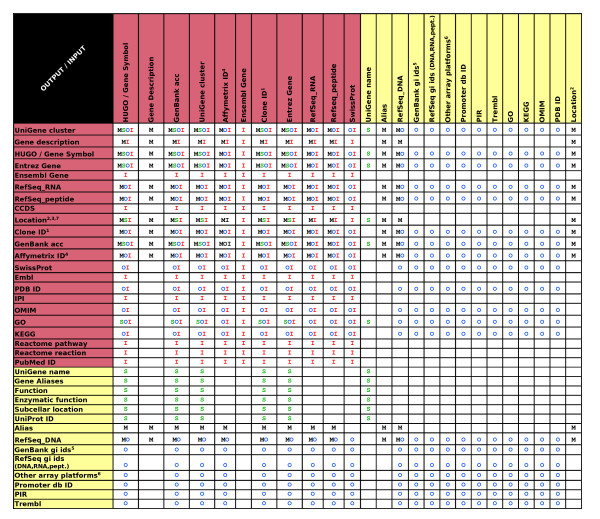
**Input and output possibilities for the four tools compared**. Description of the allowed input IDs and those IDs they can be converted to, for MatchMiner (M), SOURCE (S), Onto-Translate (O), and IDconverter (I). Notes: ^1 ^M: cDNA, FISH-mapped BAG; ^2 ^M: Cytogenetic location as input. Cytogenetic location from UCSC, transcription start and end bp; ^3 ^S: Chromosome Location, Cytoband; ^4 ^O: 18 Affymetrix arrays; ^5 ^O: dbest gi, seq id, protein gi; ^6 ^O: Agilent (5 arrays), Amersham (3), Clonetech(22), Operon (3), Perkin Elmer (5), Sigmagenosys (2), Superarray (86), Takara (6); I: Location from Ensembl and UCSC (start bp, end bp, chromosome and strand).

In Table 1, we compare the general characteristics of MatchMiner, SOURCE, and Onto-Translate with those of the two applications presented here. Also, in Figure 3, the identifiers that, according to the applications tested, can be used as input and obtained as output are shown.

**Table 1:** Comparison of conversion web applications

**Tool**	**max num of IDs entered**	**max ID types in output**	**output formats**	**extra info**
Onto-Translate [3]	no max	1	applet, text	Java applet, registration required
SOURCE Batch Search [4]	no max	all available	text	
MatchMiner Batch Lookup [5]	no max	1	HTML, text, spreadsheet, GoMiner	No rat data
IDconverter	no max	all available	HTML, text, spreadsheet	
IDClight	1	all available	HTML	

Input and output options for the different web applications compared. See Figure 3 for the description of the conversions performed by each application.

The testing procedure consisted in using several lists of identifiers (See additional file 2: Description of the test data), focusing on the time necessary for different conversions and the number of input identifiers with a corresponding output ID. Twelve different conversions have been tested for all the tools where it was possible; from four different inputs (Affymetrix HGU133A IDs, HUGO, Entrez Gene IDs, and RefSeq_RNAs) to three outputs (GenBank accessions, UniGene cluster IDs, and RefSeq_peptides). For some tools and specific conversions, the tests could not be performed successfully in any of the three rounds of testing (25th–30th August, 2006, 19th–23rd October, 2006, and 20th December, 2006) because either the application crashed or did not return any results in a reasonable time. In those cases, partial or approximated results are shown.

To ensure a fair testing process, the web servers in the U.S. (MatchMiner, SOURCE, and Onto-Translate) were tested from the authors' center in Spain; IDconverter was tested from the U.S.

#### Time performance

**Figure 4:**
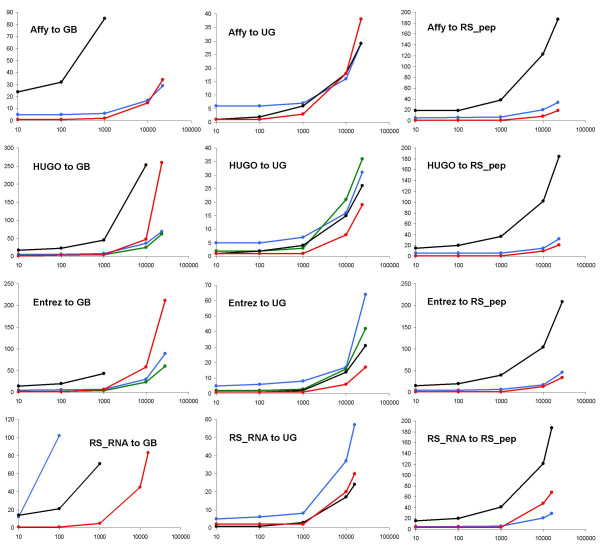
**Analysis of the time performance**. Time (in seconds) vs. number of input IDs for the twelve tests performed with MatchMiner (black lines), SOURCE (green), Onto-Translate (blue), and IDconverter (red). Abbreviations: Affy: Affymetrix ID; GB: GenBank accession; UG: UniGene cluster; RS_pep: RefSeq_peptide; Entrez: Entrez Gene ID; RS_RNA: RefSeq_RNA.

In Figure 4 the results of the time test are shown: for each conversion, a graph showing the relation between the time to perform a conversion and the number of IDs converted. For the conversions to UniGene cluster IDs, all the four tools perform similarly, but for the other conversions some differences exist. Several conclusions can be extracted from their performance:

• IDconverter was usually among the fastest applications in every conversion. Few of the large conversions took more than a minute. Those that were slower correspond to conversions from a gene identifier (like HUGO or Entrez Gene ID) to a clone identifier (such as GenBank accession).

• SOURCE, for those 4 out of the 12 conversions tested that it is built to perform, was also among the fastest tools.

• MatchMiner performed quite well for conversions to UniGene cluster, but it was slow for those to GenBank accession or to RefSeq_peptide. In some of the later cases it even crashed as it was not capable of handling the large output files.

• Onto-Translate had a fast answer time for almost all conversions, except for the conversion of RefSeq_RNAs to GenBank accessions when, even if the allotted memory to the applet was increased to 256 Mb, it was not capable of returning results for large sets and for smaller ones it took several minutes.

It has to be taken into account that two of the tools here shown, IDconverter and SOURCE, allow the user to convert to multiple types of IDs on the same batch run. However the spectrum of conversions allowed for SOURCE is smaller than that of IDconverter (Figure 3).

#### Completeness

**Figure 5:**
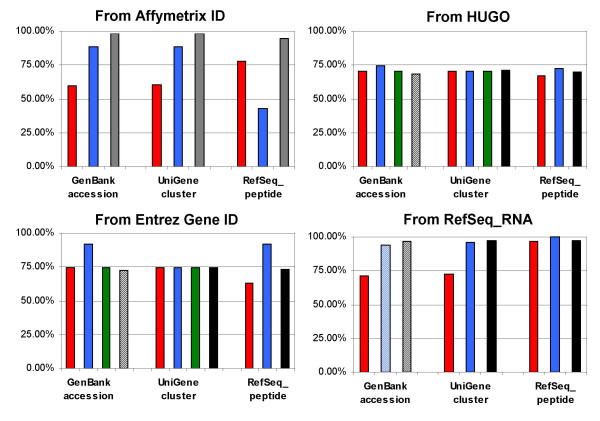
**Analysis of the completeness**. The percentage of input IDs that are converted to at least one ID is shown, for each type of input and output tested and the four applications: MatchMiner (black), SOURCE (green), Onto-Translate (blue), and IDconverter (red). Solid colors: Percentage is calculated after running the whole set through the application. Diagonal lines: The application was not able to convert the whole set, thus the percentage is taken from a smaller set. Horizontal lines: MatchMiner does not allow the user to specify to which Affymetrix array the input IDs belong, thus, given that the same probeset id can be present in different Affymetrix arrays, the percentage has to be considered an upper boundary.

In Figure 5, the percentage of input IDs with at least a converted ID is displayed for the 12 conversions timed in the previous section. These percentages were calculated using the complete sets when possible. However, in those cases were some applications were not capable of handling these large sets (bars with diagonal lines), the percentages yielded by smaller sets were used as approximations. The case of MatchMiner and Affymetrix IDs as input is different, as this application does not allow the user the specify to which array the input data belong, so when entering probeset ids from HGU 133A results from another Affymetrix array may be obtained. For this reason the percentages for the conversions of Affymetrix IDs with MatchMiner were considered as upper boundaries of the actual percentages. It also has to be noted that not all input IDs have to have a corresponding ID using other types of identifiers. Thus the goal of 100% in these graphs may not be possible.

From Figure 5, we can conclude that:

• For those conversions using HUGO or Entrez Gene IDs as input, all four applications performed similarly, with values around 70–75%, except for the conversion Entrez Gene ID to GenBank accession or RefSeq_peptide, where Onto-Translate had a few more results and the conversion of Entrez Gene ID to RefSeq_peptide, where IDconverter had a few less.

• For the Affymetrix ID conversions, the results depended on the output identifier; for two of them Onto-Translate was the most complete one, for the other it was IDconverter. As stated above, the results for MatchMiner are only upper boundaries.

• For the conversions from RefSeq_RNA identifiers, MatchMiner performed best overall. Onto-Translate reached 100% with those conversions to RefSeq_peptide. IDconverter values were around 70% for GenBank accession and UniGene cluster and close to 97% for RefSeq_peptide.

On average and taking into account only those percentages evaluated with the complete lists, MatchMiner returned an ID for 84% of the input IDs in 8 successfully evaluated conversions, Onto-Translate for 81% (11 conversions), and SOURCE (4 conversions) and IDconverter (12 conversions) for 72%.

## Conclusion

IDconverter and IDClight are a pair of tools that integrate some of the most used gene/clone/protein identifier conversions with several functional and bibliographic additional information. This is done in a easy-to-use, fast and up-to-date manner. Our commitment is to recreate the pregenerated tables every two months, following Ensembl update schedule, thus keeping all the conversions and additional information as updated as possible.

## Availability and requirements

• **Project name: **IDconverter and IDClight.

• **Project home page: ** (*) and  (†).

• **Operating system(s): **Web-based application (*,†).

• **Programming language: **Python (*,†) and PHP (*).

• **Other requirements: **Web browser (*,†).

• **License: **None (*,†).

• **Any restrictions to use by non-academics: **None (*,†).

Note: * – IDconverter; † – IDClight.

## Authors' contributions

PY designed the first version of IDconverter, including data from Ensembl and UniGene databases. AA upgraded IDconverter, created IDClight, added PubMed and UCSC databases, designed the table pregeneration process, and wrote the manuscript. AC and AA added Reactome and KEGG to the available outputs. RD-U took part on the design of both applications. All authors read, made corrections and approved the final manuscript.

## Supplementary Material

Additional File 1 Database schema showing the structure of the tables were the pregenerated data. Click here for file

Additional File 2 Description of the lists of identifiers used for testing Click here for file
