# Supplementary material for: IDconverter and IDClight: Conversion and annotation of gene and protein IDs
Source: BMC Bioinformatics. 2007 Jan 10;8:9. doi: 10.1186/1471-2105-8-9 (PMC1779800; doi:10.1186/1471-2105-8-9)
Supplement: Additional File 1 — Database schema showing the structure of the tables were the pregenerated data. [file 1471-2105-8-9-S1.pdf]

Database Schema

List of pregenerated tables

map_accession_hs
map_accession_mm
map_accession_rn
map_affy_hs
map_affy_mm
map_affy_rn
map_cloneid_hs
map_cloneid_mm
map_cloneid_rn
map_ensembl_hs
map_ensembl_mm
map_ensembl_rn
map_entrezgene_hs
map_entrezgene_mm
map_entrezgene_rn
map_refseqrna_hs
map_refseqrna_mm
map_refseqrna_rn
map_refseqpeptide_hs
map_refseqpeptide_mm
map_refseqpeptide_rn
map_swissprot_hs
map_swissprot_mm
map_swissprot_rn
map_unigenecluster_hs
map_unigenecluster_mm
map_unigenecluster_rn

map_accession_hs table

unigene varchar 64
ensemblgene varchar 64
ccds varchar 64
description varchar 255
locE_start varchar 64
locE_end varchar 64
locE_chr varchar 5
locE_band char 2
locGP_start varchar 64
locGP_end varchar 64
locGP_chr varchar 5
locGP_band char 2
hugo varchar 64
refseq_rna varchar 64
refseq_peptide varchar 64
entrezgene varchar 64
clone_id text
accession text
affy text
swissprot_name varchar 64
swissprot_accession varchar 64
embl varchar 255
pdb_id varchar 64
ipi varchar 64
go text
mim varchar 64
pmid text
keggpathid text
reactpathid varchar 255
reactpathname text
reactreacid varchar 255
reactreacname text

Note: All tables have the same fields as the one shown. When multiple identifiers map a single id, those are separated by a "|" in the record.
