# Supplementary material for: IDconverter and IDClight: Conversion and annotation of gene and protein IDs
Source: BMC Bioinformatics. 2007 Jan 10;8:9. doi: 10.1186/1471-2105-8-9 (PMC1779800; doi:10.1186/1471-2105-8-9)
Supplement: Additional File 2 — Description of the lists of identifiers used for testing [file 1471-2105-8-9-S2.pdf]

Supplementary material 2 for “IDconverter and IDClight: Conversion and annotation of gene and protein IDs”

Andreu Alibés, Patricio Yankilevich, Andrés Cañada and Ramón Díaz-Uriarte
Structural Biology and Biocomputing Programme
Centro Nacional de Investigaciones Oncológicas (CNIO)
Melchor Fernández Almagro 3, Madrid 28029, Spain
aalibes@gmail.com

Description of the test data

Four different types of identifiers were chosen to test SOURCE, MatchMiner, Onto-Translate and IDconverter. For each one, randomly sorted and selected sets of 10, 100, 1000, 10000 were used, as well as the randomly sorted complete set described below:

- **Affymetrix ID**
  - From the Affymetrix array HGU133A
- **HUGO**
  - Approved HUGO gene symbols from http://www.gene.ucl.ac.uk/nomenclature/data/gdlw_index.html
- **Entrez Gene ID**
  - Human, protein-coding Entrez Gene IDs from ftp://ftp.ncbi.nlm.nih.gov/gene/DATA/gene_info.gz
- **RefSeq_RNA**
  - Validated or Reviewed Human RefSeq_RNA starting with "NM_“ from ftp://ftp.ncbi.nih.gov/refseq/release/release-catalog/RefSeq-release18.catalog.gz

All these sets were downloaded on August 24th, 2006
These sets can be found at: http://idconverter.bioinfo.cnio.es/help/test-files.html
